# R13 preserves motor performance in SOD1^G93A^ mice by improving mitochondrial function

**DOI:** 10.7150/thno.56070

**Published:** 2021-05-24

**Authors:** Xiao Li, Chongyang Chen, Xu Zhan, Binyao Li, Zaijun Zhang, Shupeng Li, Yongmei Xie, Xiangrong Song, Yuanyuan Shen, Jianjun Liu, Ping Liu, Gong-Ping Liu, Xifei Yang

**Affiliations:** 1 Key Laboratory of Modern Toxicology of Shenzhen, Shenzhen Medical Key Subject of Modern Toxicology, Shenzhen Center for Disease Control and Prevention, Shenzhen, 518055, China.; 2 Department of Pathophysiology, School of Basic Medicine and the Collaborative Innovation Center for Brain Science, Key Laboratory of Ministry of Education of China and Hubei Province for Neurological Disorders, Tongji Medical College, Huazhong University of Science and Technology, Wuhan, China.; 3 Tianjin Institute of Pharmaceutical Research New Drug Assessment Co. Ltd, Tianjin 300301, China.; 4 Institute of New Drug Research and Guangzhou, Key Laboratory of Innovative Chemical Drug Research in Cardio-Cerebrovascular Diseases, Jinan University College of Pharmacy, Guangzhou 510632, China.; 5 School of Chemical Biology and Biotechnology, Peking University Shenzhen Graduate School, Shenzhen, 518055, China.; 6 State Key Laboratory of Biotherapy, West China Hospital, Sichuan University, and Collaborative Innovation Center for Biotherapy, Sichuan University, Chengdu, China.; 7 Department of Critical Care Medicine, State Key Laboratory of Biotherapy, West China Hospital, Sichuan University, Chengdu, China.; 8 National-Regional Key Technology Engineering Laboratory for Medical Ultrasound, School of Biomedical Engineering, Health Science Center, Shenzhen University, Shenzhen, 518060, China.; 9 Co-innovation Center of Neuroregeneration, Nantong University, Nantong, JS, China.

**Keywords:** Amyotrophic lateral sclerosis, 7,8-dihydroxyflavone, Mitochondria, Mitochondriomics, Motor performance

## Abstract

Amyotrophic lateral sclerosis (ALS) is a progressive neurodegenerative disease characterized by death of motor neurons in the brain and spinal cord. However, so far, there is no effective treatment for ALS.

**Methods:** In this study, R13, a prodrug of 7,8-dihydroxyflavone, selectively activating tyrosine kinase receptor B (TrkB) signaling pathway, was administered prophylactically to 40-day old SOD1^G93A^ mice for 90 days. The motor performance was investigated by rotarod test, climbing-pole test, grip strength test and hanging endurance test. Afterwards, the spinal cord and medulla oblongata of 130-day old mice were harvested, and the proteomics revealed the effect of R13 on mouse protein expression profile. Astrocytes and microglial proliferation were assessed by immunohistochemical analysis. The number of motor neurons in the spinal cord is determined by Nissl staining. The effect of R13 on gastrocnemius morphology was assessed by HE staining. The effect of R13 on the survival rate was accomplished with worms stably expressing G93A SOD1.

**Results:** Behavioral tests showed that R13 significantly attenuated abnormal motor performance of SOD1^G93A^ mice. R13 reduced the advance of spinal motor neuron pathology and gastrocnemius muscle atrophy. The proliferation of microglia and astrocytes was reduced by R13 treatment. Mitochondriomics analysis revealed that R13 modified the mitochondrial protein expression profiles in the medulla oblongata and spinal cord of SOD1^G93A^ mice, particularly promoting the expression of proteins related to oxidative phosphorylation (OXPHOS). Further study found that R13 activated AMPK/PGC-1α/Nrf1/Tfam, promoted mitochondrial biogenesis and ameliorated mitochondrial dysfunction. Lastly, R13 prolonged the survival rate of worms stably expressing G93A SOD1.

**Conclusions:** These findings suggest oral R13 treatment slowed the advance of motor system disease in a reliable animal model of ALS, supporting that R13 might be useful for treating ALS.

## Introduction

Amyotrophic lateral sclerosis (ALS), a progressive and uniformly fatal neurodegenerative disease, is characterized by cortical and spinal motor neuron loss, neurogenic muscular atrophy, limb weakness and eventual paralysis 1. The worldwide annual incidence of ALS is approximately 3~5 per 100,000, with fairly uniform rates and few exceptions, except for the former very high incidence of ALS on Guam 2. Latency between arrival on Guam and clinical onset of ALS often spans decades, which suggests a prolonged pathogenic process. Patients with ALS have an average age of onset of 55-60 years, a median post-diagnosis survival of 2~4 years, with death resulting from respiratory failure or pneumonia. ALS contains both sporadic (SALS) and familial ALS (FALS), with appropriate percentages of 5-10% and 90-95%, respectively 3. Approximately 20% of familial cases and 3% of sporadic cases of ALS are associated with mutations in the gene encoding superoxide dismutase-1 (SOD1) 4, but the molecular mechanisms linking this mutation to motor neuron degeneration has yet to be unsolved. There is also a lack of effective drugs for ALS treatment. Currently, riluzole and edaravone are two FDA-approved molecules for ALS, but they only modestly relieve symptoms and do not prevent disease progression 5.

Brain-derived neurotrophic factor (BDNF), a member of the neurotrophin family, could potentially play a neuroprotective role in neurodegenerative diseases, such as ALS, Alzheimer's disease, Parkinson disease and Huntington disease, among others 6, 7. As previously described, BDNF promoted motor neuron differentiation and survival 8 and prevented spinal motor neuron death from axotomy 9. Recombinant human BDNF improved motor function (grip strength and muscle weight) in the Wobbler mouse, another model of ALS, which arised from a point mutation in the Golgi-associated retrograde protein (GARP) complex 10.

BDNF mainly exerts biological functions by binding the p75 neurotrophin receptor (p75NTR) or TrkB receptor 11. The BDNF-TrkB down-signaling mechanism involves three principal tyrosine kinase pathways: PI3K-AKT pathway, MAPK-ERK (extracellular signal-regulated kinase) pathway, and PLCγ1/PKC pathway 12. BDNF-TrkB signaling improved spinal root injuries in an acute injury model of motoneuron degeneration 13, 14. Moreover, phosphorylated TrkB receptors significantly decreased in the spinal cord of ALS patients 15. However, recombinant BDNF failed to reverse motor decline or extend lifespan in both ALS mice and in a Phase III clinical ALS trial, possibly because the half-life of BDNF is too short 16.

7,8-Dihydroxyflavone (7,8-DHF) can activate the TrkB receptor and thereby mimic the physiological function of BDNF 17. Systemic administration of 7,8-DHF had been used to treat AD 18, 19, anxiety and depression 20, 21. Moreover, 7,8-DHF promoted motoneuron survival and improved motor performance in a mouse model of ALS 22, 23. However, the oral bioavailability of 7,8-DHF is not optimal. A new compound R13, a prodrug of 7,8-DHF that was reported to have better but still modest oral bioavailability and longer duration of action, activated TrkB and downstream signaling pathways, alleviated Aβ deposition and rescued memory deficits in the 5×FAD moue model of AD 24.

In this study, we explored the therapeutic efficacy of R13 in SOD1^G93A^ mice, a transgenic model of human ALS. We found that chronic oral treatment with R13 ameliorated motor performance of SOD1^G93A^ mice by improving mitochondrial functions.

## Material and methods

### Regents and antibodies

R13 (stated purity ≥ 98%) was purchased from Syntheall Pharmaceutical Company (Product name: C160505151-A, Customer ID: R13, Changzhou, China). The dilution ratios and product information of antibodies are shown in Supplementary Table 1.

### Animal treatment

Animals were purchase from purchased form Jackson Laboratory (Maine, USA), including the ALS model mouse transgenic for human SOD1^G93A^ (B6Cg-Tg (SOD1*G93A)1Gur/J, stock number 004435), which express a G93A mutant form of human SOD-1. Animals were housed in a 12-h light-dark cycle room with stable temperature (20 ± 2 ºC) and humidity (55 ± 5%). ALS animals aged 40 days were treated orally for 90 days with one of three daily doses of R13 (3.6 mg/kg (n = 9), 21.8 mg/kg (n = 10), or 43.6 mg/kg (n = 12), respectively) 24. Non-transgenic mice of gender-matched littermates (WT (n = 10)) or transgenic mice (n = 9) were treated with an equivalent volume of vehicle for 90 days. Animals were given behavioral tests when treated with R13 or vehicle for 80 days, and then terminally anesthetized.

All animal experiments were performed according to the 'Policies on the Use of Animals and Humans in Neuroscience Research' revised and approved by the Society for Neuroscience (USA) in 1995, and the Guidelines for the Care and Use of Laboratory Animals of the Ministry of Science and Technology of the People's Republic of China. The Institutional Animal Care and Use Committee at Tongji Medical College, Huazhong University of Science and Technology approved the study protocol. All the efforts were to minimize animal suffering and reduce the number of mice used.

### Behavioral tests

#### Rotarod test

This was used to assess coordination, strength and balance. Each mouse was placed on a swivel bar fatigue tester and the rotation speed set at 30 rpm. Running time on the swivel bar (at the bar time) was recorded for 5 min. All the animals were trained twice daily for 3 days after the 80 days treatment, with 300 s as the cut-off value, and then tested. The experiment was repeated three times, and the longest bar time value of each mouse was calculated as the evaluation value.

#### Climbing-pole test

Pole-climbing ability was used to evaluate the combination of motor strength and coordination 25. A homemade wooden pole (~50 cm length, ~1 cm diameter) was wrapped with gauze to promote friction. The mouse with head down was gently placed on the top of the rod, which was placed vertically on a table-top. The time taken for the mouse to descend from the top of the rod to the bottom of the platform (climbing time) was recorded. Animals were trained twice daily for 3 consecutive days after the 80 days treatment period, with 15 s as the cut-off value. The experiment was repeated three times, and the average climbing time for each mouse was recorded as the evaluation value.

### Grip strength test

Grip strength of paws was assessed to measure forelimb muscle power. Each animal was placed on the central table of the grip plate and the tail pulled gently to encourage the mouse to grasp the grip plate. When the mouse had firmly grasped the grip plate, the tail was pulled until the animal relinquished its grip. The maximum gripping power was recorded. The experiment process was repeated three times per animal after the 80 days treatment, and the largest value among the three results was taken as the evaluation value.

### Hanging endurance test

An inverted grid suspension experiment was used to evaluate the gripping power of the mouse limbs. Each animal was placed in the center of a 21 cm × 21 cm wire grid (line width of ~0.1 cm, spacing, 0.5 cm). The grid was then tapped to make the mouse grip tight, following which the grid was slowly inverted to horizontal. The time during which the mouse hung onto the grid (grip time) was recorded. Animals were trained twice daily for 3 consecutive days after the 80 days treatment, with 90 s (or longer) as the cut-off value. The experiment was repeated three times per animal, and the average hanging time value for each mouse was calculated as the evaluation value.

### Proteomics

#### Protein extraction and digestion

The medulla oblongata and lumbar 4~5 spinal cord were removed from each animal and immediately stored in -80 ºC until use. Each tissue was lysed in lysis buffer (8 M urea in 1× PBS, pH 8.0, 1× Protease and Phosphatase Inhibitor [Thermo Scientific, New Jersey, USA]) and sonicated. The lysed sample was centrifuged at 12,000 g, 4 ºC for 30 min followed by collected the supernatant. BCA protein assay kit (Thermo Fisher, NJ, USA) was used to measure the concentration of extracted protein. For spinal cord samples, a total 100 μg of protein sample was pooled from equal portions of six individual mouse samples. The pooled sample was incubated with 10 mM dithiothreitol (DTT) for 1 h at 55 ºC followed by incubation with 25 mM iodoacetamide (IAA) for 1 h at room temperature (dark environment). Samples were then diluted to 1.0 M urea concentration with 1× PBS (pH 8.0) and digested with trypsin (1:25 w/w) (Promega, WI, USA) for 15 h at 37 °C. After digestion, the sample solution was adjusted to pH 1~2, the peptide mixture desalted with a reversed-phase column (Oasis HLB, Waters, USA) and the sample dried in a vacuum centrifuge for Tandem Mass Tag (TMT) labeling. For medullary tissue, 4 individual samples per group and 50 μg protein per sample were used for protein digestion and TMT labeling as for spinal cord samples.

#### TMT labeling and LC-MS/MS analysis

Peptides from medulla or spinal cord samples were redissolved in 50 µL 200 mM triethylammonium bicarbonate (TEAB) buffer. Then peptide was labeled with TMT reagents at room temperature for 1 h and the reaction terminated by addition of 5% hydroxylamine for 15 min at room temperature. Six labeled samples including WT, ALS, ALS + 3.6 mg/kg, ALS + 21.8 mg/kg, and ALS + 43.6 mg/kg (TMT-127 to TMT-131) were mixed, desalted, dried, and re-dissolved in 100 µL 0.1% FA. Labeled peptides were loaded onto the Xbridge BEH300 C18 column (Waters, USA) for separation of peptide samples with UltiMate 3000 UHPLC (Thermo Fisher Scientific, USA) and separated into 15 fractions according to the manual instructions. All fractions were then dried, dissolved in 20 µL 0.1% FA followed by liquid chromatography (LC)-mass spectrometry (MS)/MS analysis. Briefly, the 15 fractions were separated by liquid chromatography (Acclaim PepMap100, C18, Dionex) with a 120 min flow rate at 0.30 μL/min and directly interfaced with Q-Exactive mass spectrometer (Thermo Scientific, NJ, USA). Row data were acquired by employing a single full-scan mass spectrum in the Orbitrap (400-1800 m/z, 70,000 resolution) followed by top 20 data-dependent MS/MS scans with 27% normalized collision energy. Finally, row data were searched against the database of UniProt mouse FASTA with Proteome Discoverer software. Relative protein quantification was calculated according to the reporter ion intensities of each peptide. The up and down regulation of protein expression between any two groups was set to at ratio ≥ 1.2 or ≤ 0.83.

### Bioinformatics analysis

Abundance of all differentially expressed proteins per group were estimated by heatmap analysis. R software package Mfuzz was used for protein cluster analysis to identify biological processes and pathways of expressed proteins in R13- and vehicle- treated animal groups. DAVID Bioinformatics Resources 6.8 (https://david.ncifcrf.gov/) was used for gene ontology analysis and WebGestalt (www.webgestalt.org) was searched for pathways. Molecular Complex Detection (MCODE) analysis was used to find dense regions of protein-protein interaction (PPI). Rstudio and Cytoscape software were used to visualize images.

### Western blotting

Medulla oblongata and spinal cord tissues were separately lysed ultrasonically with RIPA lysate buffer contained 1× Protease and Phosphatase Inhibitor (Thermo Scientific, NJ, USA). After protein quantification, samples were mixed with 1× loading buffer and heated for 10 min at 95 °C. Samples were separated on 8%~12% SDS-PAGE and transferred to PVDF membranes, which were then blocked for 2 h with 5% skim milk. Blocked membranes were incubated with primary antibodies overnight at 4 °C (the used antibodies information shown in Table S1). After washing in TBST, membranes were incubated with proper dilution of secondary antibody for 1 h at room temperature. After exposure using an ECL kit (Thermo Scientific, NJ, USA), target protein quantitative densitometry analysis was performed with ImageJ software.

### Hematoxylin-eosin and Nissl staining, immunohistochemistry and immunofluorescence

Lumbar 4~5 spinal cord samples were isolated, fixed with 4% paraformaldehyde for 48 h, dehydrated stepwise in gradient sucrose and embedded in paraffin. Sections (5 μm) of spinal cord were stained with hematoxylin-eosin or cresyl violet for pathological examination. For immunohistochemistry and immunofluorescence, sections were boiled for 10 min with sodium citrate for antigen retrieval followed by incubating with primary antibody GFAP, Iba1, CHAT, or TrkB in 0.3% Triton X-100 phosphate buffer saline (PBS) overnight at 4 °C. Rabbit-specific HRP/DAB (ABC) Detection IHC Kit was used for immunohistochemistry. Briefly, after incubation with the primary antibody, sections were incubated with biotinylated goat anti-polyvalent for 10 min and washed with PBS, streptavidin peroxidase for another 10 min and washed with PBS, DAB diluent for 2-5 min and washed with water. Finally, sections were stained with hematoxylin, dehydrated with 100-70% gradient ethanol and cleared with xylene. For immunofluorescence, after incubated with primary antibody, sections were washed with PBS followed by incubation with fluorescent secondary antibody 488 or 568 and washed with PBS. Finally, sections were stained with DAPI, washed with PBS and covered with film. All histological sections were examined with a light microscope and analyzed with Image-pro plus 6.2 software.

### Mitochondrial functional analysis

Mitochondrial function of the medulla oblongata and spinal cord was evaluated by determining levels of lipid peroxidation and ATP according to previously published methods 26.

### Lipid peroxidation assay

The Lipid Peroxidation MDA Assay Kit (Beyotime, Haimen, China) was used to measure malondialdehyde (MDA) production in CNS tissues from each group of mice. Briefly, freshly extracted samples were measured for protein concentration, and then samples (100 µL) were incubated with MDA detection fluid (200 µL) at 100 °C for 15 min, and centrifuged at 1,000 g for 10 min. Finally, 200 µL of the supernatant was added to a 96-well plate and the absorbance measured at 532 nm. The lipid peroxidation level was calculated as nmol/mg protein.

### ATP levels

The ATP level of medulla oblongata and spinal cord tissues was measured with an ATP kit (Beyotime, Haimen, China). Briefly, after protein extraction and measurement, CNS tissue samples (100 µL) were mixed with ATP detection fluid (100 µL) and incubated at room temperature for 3 min. The ATP content was read by a microplate reader with luminometer function. ATP levels were calculated as nmol/mg protein.

### *C. elegans* transgenic strains and lifespan assay

C. elegans were raised on Nematode Growth Medium (NGM) plates seeded with OP50 E. Coli at 20 °C. The strains used were N2 Bristol, plEx102[Punc-47::SOD1::SL2::mStrawberry, Pmyo-2::mStrawberry], and plEx104[Punc-47::SOD1^G93A^::SL2::mStrawberry, Pmyo-2:: mStrawberry]. To generate the transgenic strains expressing human wild type SOD1 or mutant SOD1^G93A^ in D-type motor neurons of C. elegans, we created two plasmids that include the unc-47 promoter and cDNA of SOD1 or SOD1^G93A^ followed by a spliced leader SL2 and mStrawberry (Punc-47::SOD1::SL2::mStrawberry and Punc-47::SOD1^G93A^::SL2::mStrawberry). SOD1 cDNA and SOD1^G93A^ cDNA were amplified from addgene plasmids #26407 and #26411, respectively. The cDNA region of newly generated plasmids was confirmed by sequencing. Microinjections were performed following standard protocol. Each plasmid DNA in the transgenic strains was injected into N2 Bristol at a concentration of 25 ng/μL. Two independent transgenic lines of each strain were generated and tested for lifespan to confirm the results, while only the data from one transgenic line were shown for simplicity and clarity.

Lifespan experiments were performed on NGM plates with a layer of OP50 either without or with 7,8-DHF at a concentration of 0.5 mg/mL or 2 mg/mL at 20 °C. Transgenic worms were identified by mStrawberry expression. For each lifespan assay, about 60-80 worms were included and transferred every other day to fresh NGM plates at a density of 15 worms per plate. The first day of adulthood was scored as day 1. Survival was scored every day, and the worms that crawled off the plate, hatched inside, or exploded were censored. Statistical analyses were performed using Prism 8 (GraphPad). p values were calculated using the log-rank (Kaplan-Meier) method.

### Statistical analysis

Data were expressed as the Mean ± SEM (standard error). Statistical analysis was performed using either an ANOVA (equal variance) or a Welch's ANOVA (unequal variance) test followed by Bonferroni's post-hoc test with SPSS 21.0 software. The significant difference among the groups was set at p < 0.05.

## Results

### Administration of R13 ameliorated the motor performance of SOD1^G93A^ mice

**Figure 1:**
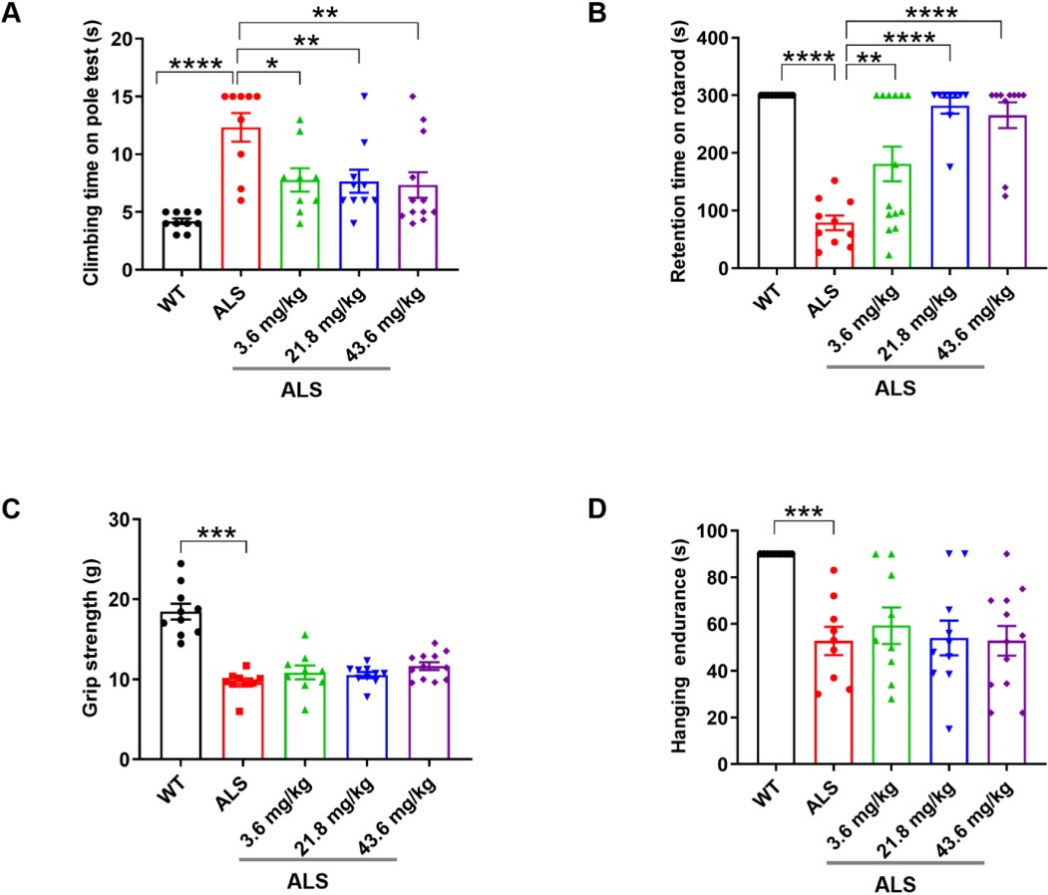
** Preventive R13 administration improved motor performances of SOD1^G93A^ mice.** 40-d old SOD1^G93A^ mice were administrated with R13 for 80 days, and then, motor performance detected, **(A)** climbing time on pole tests by climbing-pole test,** (B)** retention time on rotarod by rotarod test, **(C)** grip strength measured by grip strength test and** (D)** hanging endurance time detected with hanging endurance test. Data was shown Mean ± SEM. *, *p* < 0.05, **, *p* < 0.01, ***, *p* < 0.001, ****, *p* < 0.0001. n = 10-14 for each group.

We orally administered R13 for 90 days to young adult (40-d old) SOD1^G93A^ transgenic ALS model mice over a dose range of 3.6~43.6 mg/kg, with SOD1^G93A^ model and WT mice receiving an equivalent daily volume of vehicle for the same period. R13 and vehicle administration started at 40 d old pre-symptomatic mice, and ended at 120 d of age, during which motor dysfunction was progressively evident. The prophylactic drug treatment scheme was based on the standard research protocol established for the SOD1^G93A^ mutant mouse model 27. Firstly, we showed that oral administration of R13 did not cause weight changes in mice at the highest dose (Figure S1). Performance on behavioral tests, namely the rotarod, climbing pole, grip strength and wire hanging method, was used to evaluate the motor ability of ALS mice as the disease progressed, after treated with R13 for 80 days 28, 29. Compared with the performance of control animals, ~120 d-old ALS model mice showed decreased performance on motor function tests (Figure 1). R13 treatment significantly raised the behavioral performance of SOD1^G93A^ mice compared with controls on the rotarod and climbing pole tests (Figure 1A-B), while the grip strength and hanging endurance time showed no significant change (Figure 1C-D). These data suggest that R13 treatment selectively protected complex motor functions of SOD1^G93A^ mice without detectable changes in paw strength.

### Administration of 7,8-DHF increased median survival of SOD1^G93A^ worms

Because R13 was stable in an acidic environment, it was not suitable to use R13 to treat transgenic worms. Therefore, we treated transgenic worms with 7,8-DHF (active ingredient R13) to explore its impact on the survival rate. We found that the median survival of worms stably expressing G93A SOD1 was remarkably declined compared to N2 worms (16.067±1.106 versus 21.170±0.720 days; P < 0.0001, Figure S2A) or worms stably expressing wt SOD1 (16.067±1.106 versus 20.563±0.713 days; P < 0.01, Figure S2A). However, treatment with 2 mg/mL 7,8-DHF resulted in significantly increased median survival of worms stably expressing G93A SOD1 compared to vehicle-treated control worms (19.625±0.738 versus 16.067±1.106 days; P < 0.001, Figure S2A). But 7,8-DHF have no effect on the paralysis rate of worms stably expressing G93A SOD1 (Figure S2B). These results indicate that 7,8-DHF significantly prolonged the lifespan of SOD1^G93A^ transgenic worms.

### R13 modified the protein expression profile in SOD1^G93A^ mice

First, non-targeted proteomics and TMT labeling were used to characterize the protein expression change. With FDR < 0.01, a total of 5767 proteins was identified and quantified in spinal cord samples, and 4740 proteins were found in medulla oblongata. Compared with 130 d-old WT mice, there were 336 differentially expressed (DE) proteins in spinal cord from 130 d-old SOD1^G93A^ model mice (Figure S3A). Bioinformatic analysis found up-regulation proteins enriched biological process in transport, chemical synaptic transmission, astrocyte development, etc. (Figure S3B). And corresponding pathway enriched in spinal cord injury, synaptic vesicle cycle, oxidative damage, microglia pathogen phagocytosis pathway, etc. (Figure S3D). The down-regulation proteins mainly enriched in muscle contraction, regulation of ATPase activity, etc. (Figure S3C) and pathways enriched in striated muscle contraction, cardiac muscle contraction, focal adhesion, glycolysis and gluconeogenesis, etc. (Figure S3E).

In medulla oblongata, compared with WT mice, 226 differentially expressed proteins was found in ALS mice (Figure S4A) and the up-regulation proteins enriched biological process in innate immune response, cell-cell adhesion, regulation cell shape, etc. (Figure S4B), while pathway also included oxidative damage, microglia pathogen phagocytosis pathway and others (Figure S4D). The down-regulation proteins involved in regulation membrane potential, cellular aldehyde metabolic process, transport, etc. (Figure S4C), and pathways focused in metabolism of xenobiotics by cytochrome P450, glycolysis and gluconeogenesis (Figure S4E). From the analysis, we found that ALS mice showed disorder in synaptic function, glial proliferation and immune inflammatory response, muscle contraction, glycolysis and gluconeogenesis. The spinal cord and medulla oblongata showed common disorders in glycolysis and gluconeogenesis, microglia pathogen phagocytosis. All these, at least, showed energy metabolism disorder which was reflected by mitochondrial dysfunction in ALS mice. So, improvement of mitochondrial function was an approach to ameliorate ALS disease.

**Figure 2:**
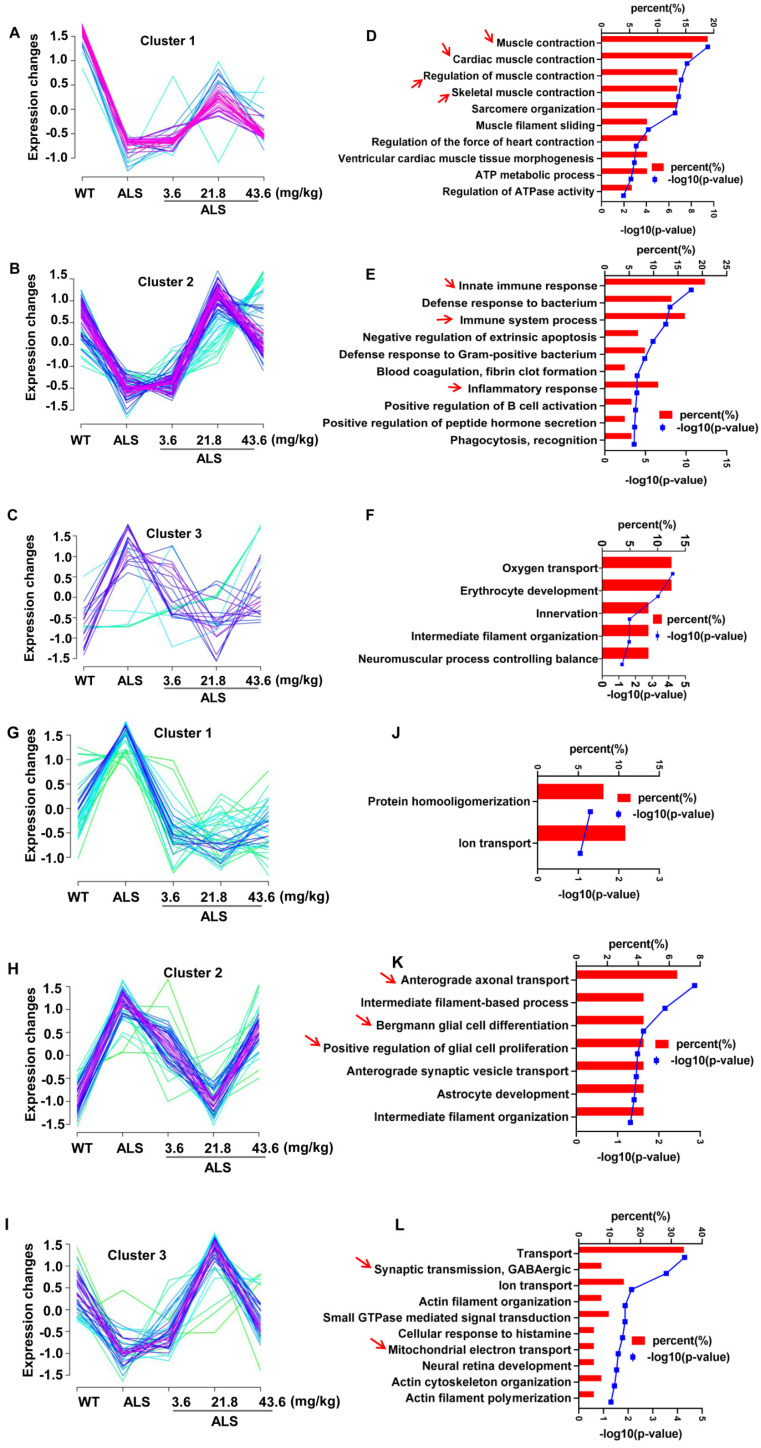
** Cluster analysis showed the 3 ameliorated patterns in different doses of R13 treatment and corresponding biological process enriched by Gene ontology analysis.** Three ameliorated expression patterns **(A-C)** and corresponding top 10 biological processes **(D-F)** with enriched p value less than 0.05 in proteomic of spinal cord. Three ameliorated expression patterns **(G-I)** and corresponding top 10 biological processes **(J-L)** with enriched p value less than 0.05 in proteomic of medulla oblongata. Arrows indicated highly enriched score and more meaningful biological processes.

Compared with SOD1^G93A^ model mice, we acquired a total 144 DE proteins in spinal cord (Figure S5A) and 207 DE proteins in medulla oblongata (Figure S5B) induced by R13 administration via differential expression analysis (Table S2), an illustration of which is also shown by heatmap analysis (Figure S5B). A total 144 DE proteins in lumbar spinal cord (Figure 2A-C) and 207 DE proteins in medulla oblongata (Figure 2G-I) are respectively divided into three categories through cluster analysis. Compared with 130 d-old WT mice, the protein expression in the 130 d-old SOD1^G93A^ identified associations with (a) decreased muscle contraction, immune response, synaptic transmission and mitochondrial function, and (b) increased glial cell proliferation and ion transport. R13 treatment modified the expression pattern of these proteins. Compared with the 130 d-old ALS model, cluster 1 and 2 of spinal cord showed a trend toward an increase of proteins involved in muscle contraction, cardiac muscle contraction, immune response regulation of muscle contraction, skeletal muscle contraction (Figure 2D), innate immune response, immune system process and inflammatory response, among others (Figure 2E). Cluster 2 of the medulla oblongata showed a trend toward a decrease in the enriched function in anterograde axonal transport, Bergmann glial cell differentiation and positive regulation of glial cell proliferation, etc. (Figure 2K). Cluster 3 of the medulla oblongata trended toward an increase in enriched function in transport, synaptic transmission, GABAergic and mitochondrial electron transport, etc. (Figure 2L). DAVID functional analysis noted the increased proteins that were modified by R13 in the spinal cord were mainly involved in the biological processes of muscle contraction, immunity and inflammation, while the increased proteins modified in the medulla oblongata were involved in the biological processes of synaptic transmission and mitochondrial function, while the decreased proteins were mainly involved in glial cell proliferation signaling pathway.

**Figure 3:**
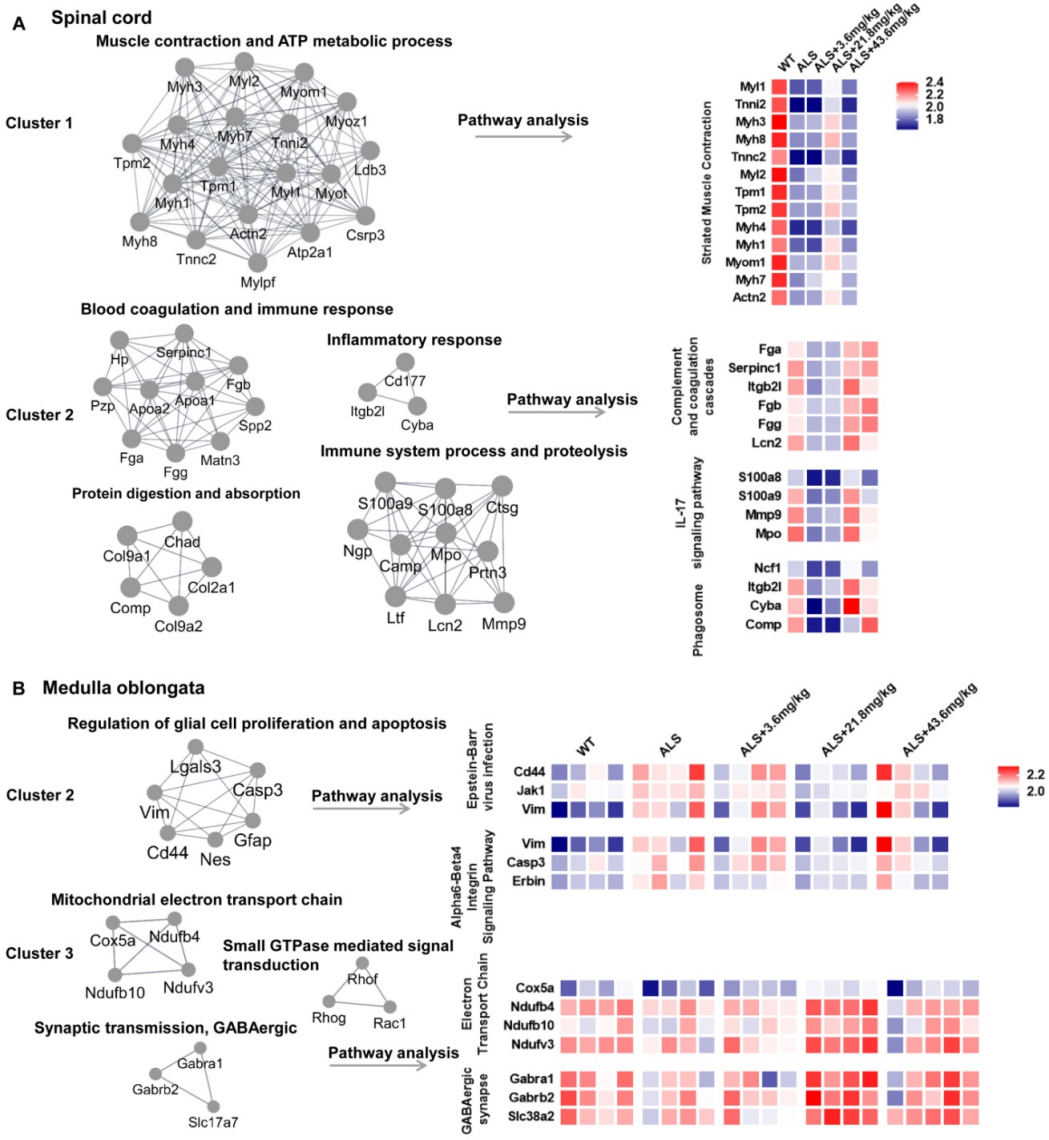
** Molecular COmplex Detection (MCODE) analysis to find the core modules in Protein-protein interaction (PPI) network followed by functional annotation and pathway analysis by WebGestalt in 2 main ameliorated clusters. (A)** The core modules and enriched pathways of cluster 1 and cluster 2 in proteomic result of spinal cord. **(B)** The core modules and enriched pathways of cluster 2 and cluster 3 in proteomic result of medulla oblongata. The color of blue represents low abundance, red represents high abundance.

To further clarify the biological significance of different cluster proteins induced by R13, we used Molecular Complex Detection (MCODE) analysis to seek the core DE protein gene set after R13 administration. For spinal cord, the core gene set of cluster 1 was associated with muscle contraction and ATP metabolic process and enriched pathway in striated muscle contraction. Cluster 2 was associated with blood coagulation and immune response, protein digestion and absorption, immune system process and proteolysis, inflammatory response and enriched pathways in complement and coagulation cascades, IL-17 signaling pathway, and phagosome (Figure 3A). For medulla oblongata, core gene set of cluster 2 was functionally in regulation of glial cell proliferation and apoptosis, and associated pathway was Epstein-Barr virus infection and Alpha6-Beta4 integrin signaling pathway. While cluster 3 was functionally in mitochondrial electron transport chain, synaptic transmission, GABAergic, small GTPase mediated signal transduction, and corresponding enriched pathways involved electron transport chain and GABAergic synapse (Figure 3B). From the overall differentially expressed proteins, we classified the R13-induced modified DE proteins in SOD1^G93A^ mice and found that the increased proteins in spinal cord were associated with muscle contraction, immunity and inflammation, while increased proteins in medulla oblongata were associated with synaptic transmission, mitochondrial function and the decreased proteins were functionally in proliferation and differentiation of glial cells.

### R13 improved mitochondrial function in SOD1^G93A^ mice

We used venny analysis to compare overlapping DE proteins in R13-treated animals as a function of dose (Figure S6B). Dose-dependent changes in the expression of mitochondrial proteins were apparent in both spinal cord and medulla oblongata tissues, which included mitochondrial fission factor, (mitochondrial) aspartate--tRNA ligase (Figure S6A), (mitochondrial) 2-methoxy-6-polyprenyl-1,4-benzoquinol methylase, and Cytochrome c (Figure S6B). Moreover, the R13- modified cluster 3 proteins include Ndufv3 (NADH dehydrogenase [ubiquinone] flavoprotein 3, mitochondrial), Ndufb4 (NADH dehydrogenase [ubiquinone] 1 beta subcomplex subunit 4), and Ndufb10 (NADH dehydrogenase [ubiquinone] 1 beta subcomplex subunit 10), cox5a (Cytochrome c oxidase subunit 5A, mitochondrial) of medulla oblongata enriched in electron transport chain were all up-regulated (Figure 3B). These data suggest that R13 may act to preserve the integrity of mitochondria.

**Figure 4:**
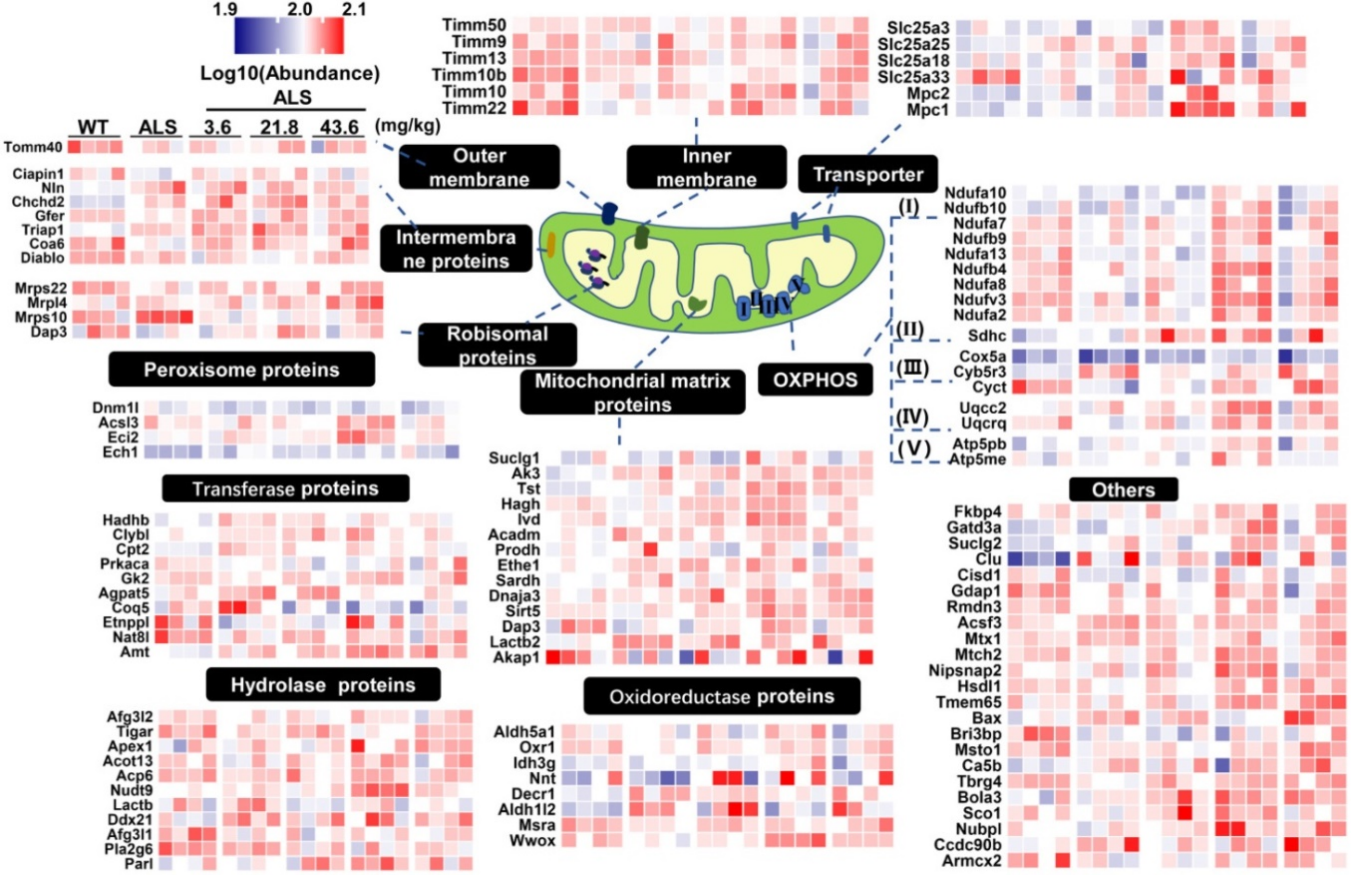
** Differential expression profile of mitochondria in proteomic result of medulla oblongata.** According to biological functions of GO annotations and uniprot database, differentially expressed proteins localized to mitochondria in medulla oblongata were mapped to peroxisome proteins, transferase proteins, hydrolase proteins, outer membrane, intermembrane proteins, ribosomal proteins, inner membrane, mitochondrial matrix proteins, oxidoreductase proteins, transporter, OXPHOS, others. The differentially expressed proteins was set as the p value of the pairwise comparison group less than 0.05. The heatmap was color coded according to the log_10_ abundance for proteins. The color of blue represented low abundance, red represented high abundance.

Expression of mitochondrial proteins in medulla oblongata was further analyzed by heatmap and localization analysis of proteins with p values < 0.05 in the pairwise comparison (Figure 4). From the mitochondrial expression profile, we found most DE mitochondrial proteins showed a tendency to up-regulate in medium-dose R13 treatment. This was especially evident for proteins involved in the electron transport chain, mitochondrial matrix, peroxisomes, transporters and other categories. Similarly, the heatmap of mitochondrial protein expression in spinal cord in a pairwise comparison ratio ≥ 1.2 or ≤ 0.83 is shown in Supplementary Figure 7. Compared with vehicle-treated SOD1^G93A^ mice, R13 treatment ameliorated levels of most of the mitochondrial protein expression of SOD1^G93A^, similar to those of WT mice. Taken together, the various foreging analyses suggest that R13 administration had a protective effect on mitochondria.

**Figure 5:**
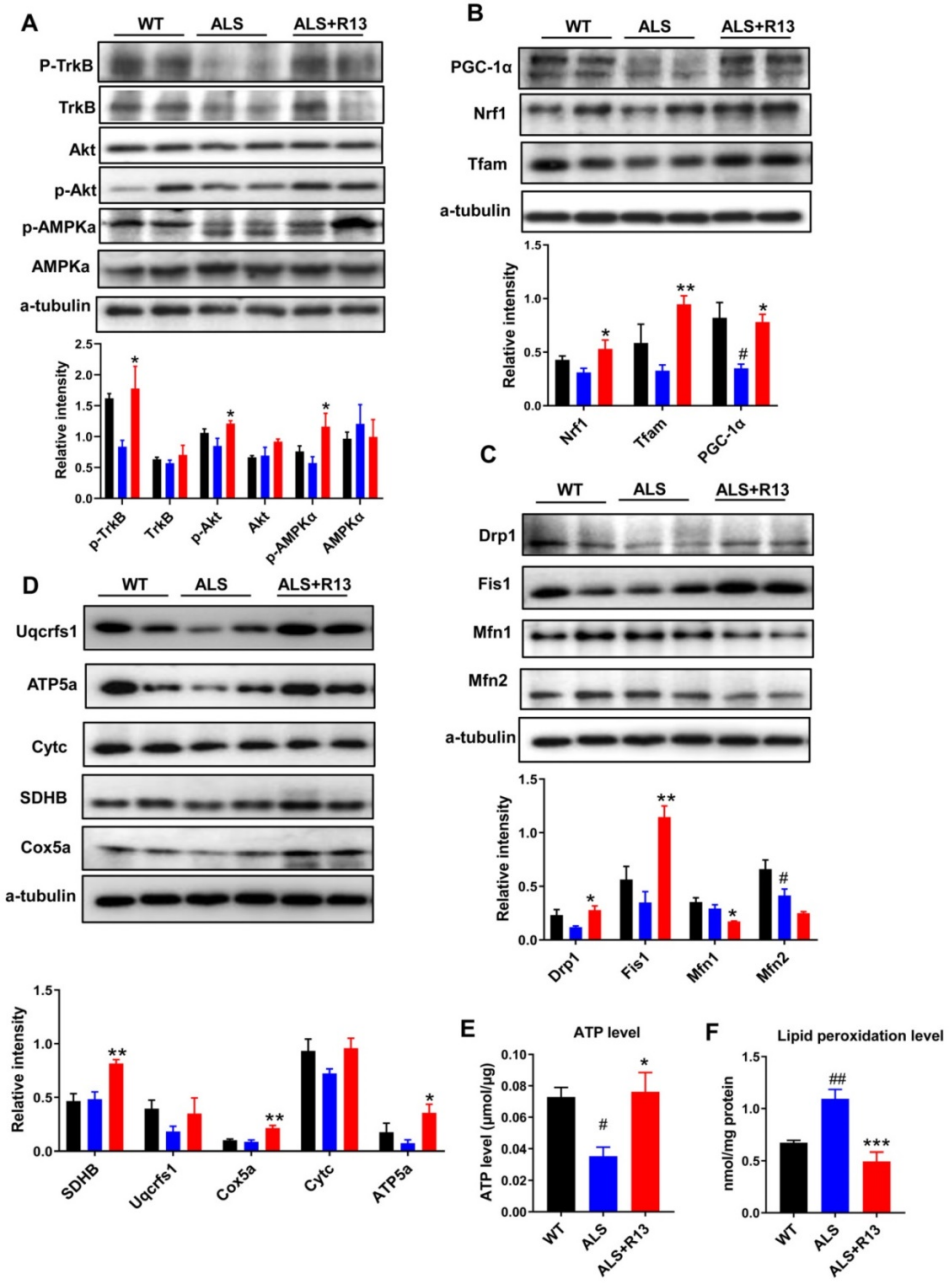
** R13 administration activated TrkB, and ameliorated mitochondrial functions by promoting mitochondrial biogenesis and change mitochondrial dynamic in medulla oblongata. (A)** Quantified the kinase active state of TrkB, Akt and AMPKα. **(B)** The expression level and quantified the proteins of mitochondrial biogenesis pathway, such as PGC-1α, Nrf1 and Tfam. **(C)** The mitochondrial dynamic proteins of drp1, fis1, mfn1 and mfn2 were detected by Western blotting and quantitative analysis. **(D)** The electron transport chain proteins of Uqcrfs1, ATP5a, Cytc, SDHB and Cox5a were detected by Western blotting and quantitative analysis. (E, F) ATP level and lipid peroxidation level were detected. Data was shown Mean ± SEM. *, *p* < 0.05, **, *p* < 0.01, ***, *p* < 0.001 vs. ALS vehicle group. #, *p* < 0.05, ##, *p* < 0.01 vs. WT group. n = 4 for each group.

We found that the functions of 21.8 mg/kg R13-induced expression change of mitochondrial proteins corresponded to mitochondrial biogenesis and mitochondrial dynamics, which control mitochondrial functional quality 30. Here, we also found that R13 treatment (21.8 mg/kg) activated the mitochondrial upstream regulator of AMPK in medulla oblongata (Figure 5A), and increased expression of proteins in the downstream pathway, including mitochondrial fission proteins (Dynamin-1-like protein (Drp1), mitochondrial fission 1 protein (Fis1) (Figure 5C), and proteins of mitochondrial biogenesis pathway (Peroxisome proliferator-activated receptor gamma coactivator 1-alpha (PGC-1α), Nuclear respiratory factor 1 (Nrf1), Transcription factor A, mitochondrial (Tfam) (Figure 5B) with increased ATP level and decreased MDA production (Figure 5E-F). In agreement with the data for mitochondrial biogenesis, levels of electron transport chain proteins, such as succinate dehydrogenase (SDHB), cytochrome c oxidase subunit 5A (Cox5a), and ATP synthase subunit alpha (ATP5a), were significantly increased in the medulla oblongata (Figure 5D). The decreased mitochondrial protein levels in spinal cord of SOD1^G93A^ mice were also ameliorated by R13 treatment (Figure S7). Western-blot analysis also showed significantly increased the mitochondrial biogenesis protein PGC-1α, mitochondrial dynamic protein Fis1 (Figure S8A), mitochondrial electron transport chain protein SDHB and Cox5a in spinal cord of SOD1^G93A^ with 21.8 mg/kg R13 vs. vehicle (Figure S8B), and dynamic protein Mfn1 decreased (Figure S8A). The ATP level of SOD1^G93A^ model was markedly decreased compared with WT in spinal cord, while R13 treatment increased the ATP level of SOD1^G93A^ mice (Figure S8C). Taken together, the data show that R13 attenuated mitochondria dysfunction in SOD1^G93A^ mice.

### R13 treatment ameliorated the pathological changes of SOD1^G93A^ mice

**Figure 6:**
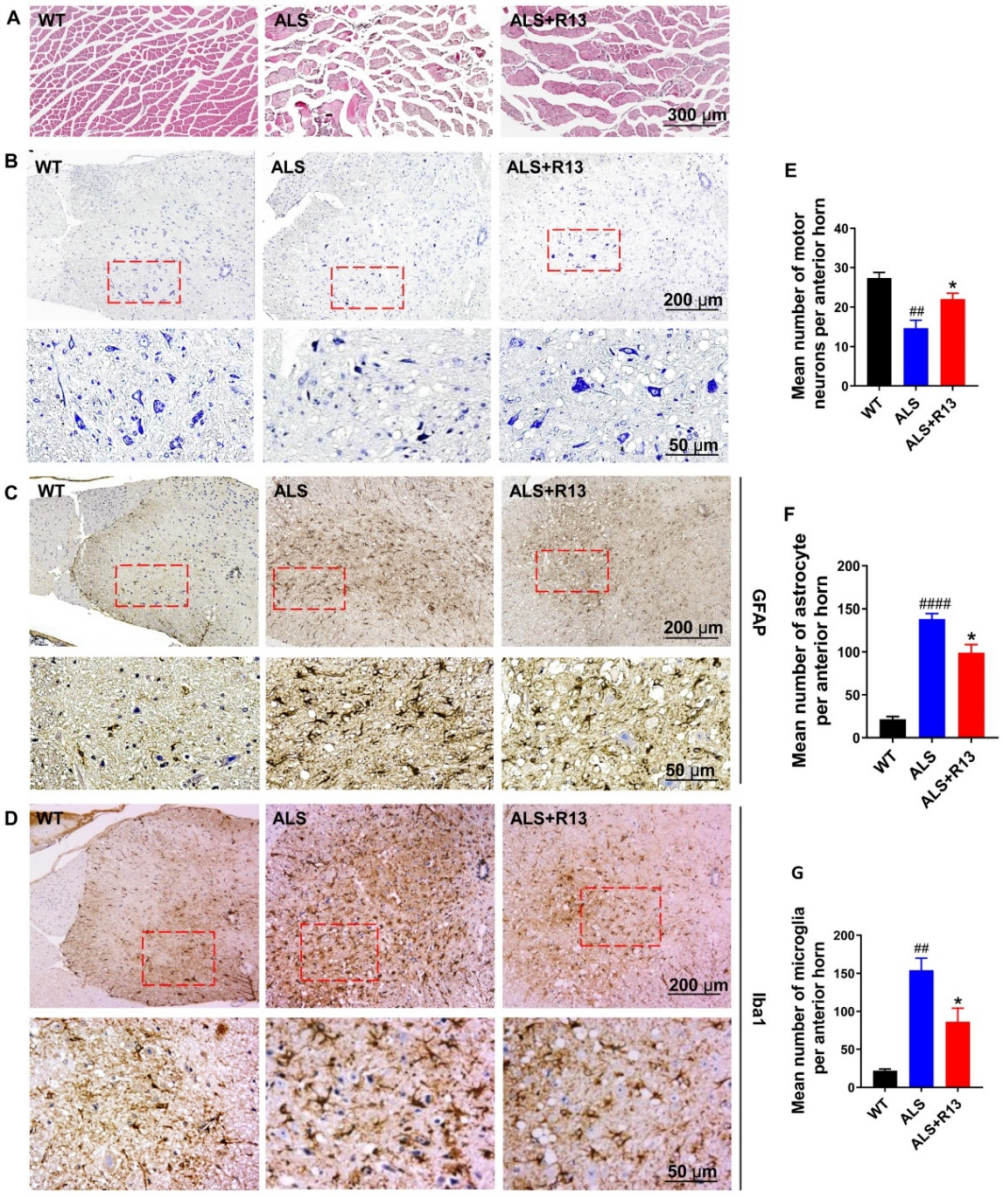
** R13 administration ameliorated gastrocnemius cross-sectional area, increased number of spinal cord motor neurons, and decreased astrocytes and microglia numbers in spinal cord of SOD1^G93A^. (A)** The representative graph of gastrocnemius cross-sectional area by H&E staining. **(B, E)** The representative image of motor neuron in spinal cord by Nissl staining and quantitative analysis. **(C, D, F, G)** The representative image of positive immunostaining of GFAP (astrocytes) and Iba1 (microglia) in spinal cord and quantitative analysis. Data was expressed as Mean ± SEM. *, *p* < 0.05 vs. ALS vehicle group; *##*, *p* < 0.01, ####, *p* < 0.0001 vs. WT group. n = 3 for each group.

Proteomics data for spinal cord showed that muscle contraction and immune response-related proteins increased, while medullary gliosis-related proteins decreased in R13-treated vs. -untreated SOD1^G93A^ (Figure 3). Immunofluorescence showed that the TrkB receptor was localized in spinal motor neurons (co-localization of TrkB receptor and CHAT) (Figure S9). R13 significantly preserved the cross-sectional area of gastrocnemius muscle (Figure 6A) and the number of neurons in spinal cord (Figure 6B, E). We also found that R13 activated TrkB and the downstream signaling pathway by increased p-TrkB and p-Erk levels in the motor cortex of ALS mice (Figure S10).

Astrocyte had toxic effects on motor neurons in patients with familial and sporadic ALS 31, and glial cell proliferation and microglial activation were one of the main characteristics of neuroinflammation in ALS 32. We used immunohistochemistry to detect the expression of astrocyte (GFAP) and microglia (Iba1) in the spinal cord. Compared with control WT mice, the number of astrocyte and microglia were significantly increased in the spinal cord of SOD1^G93A^ mice. R13 treatment decreased the positive immunostaining of GFAP (Figure 6C, F) and Iba1 (Figure 6D, G) in spinal cord of SOD1^G93A^ mice. All these results suggest that treatment of R13 reduced the degree of pathological change associated with ALS.

## Discussion

ALS is a progressive neurodegenerative disease characterized by the death of motor neurons in the brain and spinal cord, gradually progressing to generalized muscle atrophy and dysphagia, and finally respiratory failure leading to death. However, so far, there is no effective drug for the treatment of ALS. In the present study, we found that R13, a prodrug that improved the oral bioavailability of 7,8-DHF and maintained an effective concentration of 7,8-DHF in the brain for longer periods 24, ameliorated some aspects of the motor performance of SOD1^G93A^ mice. R13 treatment (a) slowed the progression of motor system dysfunction of SOD1^G93A^, (b) prolonged the survival rate of worms stably expressing G93A SOD1, (c) reduced development of the abnormal protein profile of these animals in the medulla oblongata and lumbar spinal cord, (d) reduced the loss of spinal motor neurons, and (e) preserved the cross-section area of gastrocnemius muscle, and (f) reduced the degeneration-associated microglial proliferation and astrogliosis. By the way, R13 significantly ameliorated motor system decline and related pathological changes.

Mitochondria played a key role in intracellular energy production, calcium homeostasis, and apoptosis control. Studies on postmortem tissues of ALS patients and animal models showed that the activity of the mitochondrial electron transport chain complex was reduced 2. Swollen and vacuolated mitochondria are markedly increased in spinal motor neurons of ALS patients or primary motor neurons expressing mutant SOD1 33, 34. Mitochondrial distributions in motor neuron axons were distinctly disturbed in the mice expressing mutant SOD1 35. Mitochondrion has been got more and more attentions and was considered to be an important and potential therapy target for ALS. Here, we found that R13 treatment reversed the decreased ATP and increased MDA level in the spinal cord and medulla oblongata of ALS mice, which indicated that R13 improved mitochondrial function. Moreover, proteomics revealed that R13 treatment restored the expression level of OXPHOS complex-related proteins in spinal cord and medulla oblongata. We also found that the expression levels of the mitochondrial complexes complex II (SDHB) and complex IV (Cox5a) in the spinal cord and medulla oblongata were significantly increased by R13 treatment. Moreover, complex V (ATP5a), complex IV (Cox5a) and complexes complex II (SDHB) level also were remarkably upregulated in the medulla oblongata. These results suggest that R13 may enhance the expression level of OXPHOS related proteins in ALS mice. PGC-1α is the main regulator of mitochondrial biogenesis, which improved the motor neuron function and survival rate of SOD1^G93A^ mice 36. R13 treatment increased the expression level of PGC-1α in the medulla oblongata and spinal cord tissues of ALS mice. These results suggested that R13 treatment ameliorated mitochondrial function through increasing mitochondria biogenesis. By the way, mitochondrial fission protein Fis1 also increased treatment by R13, which suggested that R13 may improve mitochondria function by promoting mitophagy.

The previous study showed that knockout of BDNF truncated receptor TrkB.T1 delayed the progression of ALS mice model 37, which may be one of the reasons for the failure of BDNF experiments in clinical ALS patients. There are three forms of TrkB receptor: full-length TrkB receptor (TrkB.FL), truncated TrkB receptor (TrkB.T1) and truncated TrkB receptor (TrkB.T2). The intracellular domains of TrkB.T1 and TrkB.T2 lack the tyrosine kinase signal domain and cannot undergo autophosphorylation. Moreover, TrkB.T1 binding to BDNF indirectly inhibits the downstream pathways of TrkB.FL including Akt and ERK/MAPK 7. A recent study has shown that R13 compound promoted TrkB autophosphorylation and its downstream signal Akt and ERK by binding to TrkB.FL 17. All the above information suggested that R13 bound with TrkB.FL is necessary to activate the downstream signaling pathway.

To investigate the mechanisms underlying R13-improved motor performance, the gastrocnemius muscle area and the number of spinal anterior horn neurons were detected. Due to the poor knowledge of R13 in ALS treatment so far, proteomic analysis was used to detect protein profiles in medulla oblongata and spinal cord. We found that a large number of proteins, including sarcoplasmic/endoplasmic reticulum calcium ATPase 1 (Atp2a1), and the proteins involving in the contraction of muscle, were up-regulation in spinal cord by R13 treatment. Here, the proteins enriched in the striated muscle contraction signaling pathway, especially the muscle proteins which playing an important role in the contraction of the gastrocnemius muscle in spinal cord, are up-regulated via TrkB activation by R13. The BDNF/TrkB signaling is one of the most implicated pathways in the neuromuscular junction stability and it is essential for neurotransmission 38. TrkB receptor also exists in the muscle 39. R13 activated TrkB in the cortex (Figure S10), spinal cord and muscle fiber to maintain the BDNF/TrkB signal pathway, which played an important role in ameliorating muscle contraction 38, 40. The increased motor neurons also help to improve the motor ability of SOD1^G93A^ mouse by R13 administration.

After treatment with R13, Microglia numbers significantly decreased, which may be due to improved mitochondrial functions in microglia by activation of TrkB. In addition, a previous study reported an unrecognized possible role of neuronal mitochondria in the regulation of microglial activation, and proposed neuronal Mfn2 as a likely mechanistic linker between neuronal mitochondria dysfunction and neuroinflammation in neurodegeneration 41. So improved neuronal mitochondrial function may also contribute to the decreased microglia. Recent studies reported that BDNF-TrkB signaling pathway was associated with inhibition of astrogliosis 42, 43. Moreover, attenuated astrocyte activation accompanied with mitochondrial dysfunction by TBN treatment in ALS mice 44, which is consistent with our findings, but the mechanisms are still unclear, waiting for further study. By the way, as R13 did not influence measures of grip strength, which is a gold-standard in ALS, this may imply that the R13 treatment may have restricted effects on motor performance in this model.

## Conclusions

In summary, R13 treatment activated AMPK to promote mitochondria biogenesis and fission via stimulating TrkB receptors and escalates mitochondrial proteins expression with increased ATP levels and decreased MDA. R13 also suppressed the proliferation of glial cells, attenuated motor neurons loss, and ameliorated gastrocnemius atrophy, possibly by upregulating the proteins involving in the striated muscle contraction pathway. Conceivably, all of these contributed to improve the motor ability of SOD1^G93A^ mice (Figure S11). Clearly, these experimental observations in a valid murine model of ALS support that R13 may be further explored for possible therapeutic use in ALS.

## Supplementary Material

Supplementary figures.

Supplementary tables.

## Abbreviations

ALS: Amyotrophic lateral sclerosis
ATP5a: ATP synthase subunit alpha
BDNF: Brain-derived neurotrophic factor
Cox5a: cytochrome c oxidase subunit 5A
Cytc: cytochrome c
Drp1: Dynamin-1-like protein
7,8-dihydroxyflavone: 7,8-DHF
Fis1: mitochondrial fission 1 protein
MCODE: Molecular Complex Detection
Ndufb4: NADH dehydrogenase [ubiquinone] 1 beta subcomplex subunit 4
Ndufb10: NADH dehydrogenase [ubiquinone] 1 beta subcomplex subunit 10
Nrf1: Nuclear respiratory factor 1
OXPHOS: oxidative phosphorylation
p75NTR: p75 neurotrophin receptor
PGC-1α: Peroxisome proliferator-activated receptor gamma coactivator 1-alpha
SDHB: succinate dehydrogenase
SOD1: superoxide dismutase-1
Tfam: Transcription factor A, mitochondrial
TrkB: Tyrosine kinase receptor B
