# Supplementary material for: R13 preserves motor performance in SOD1G93A mice by improving mitochondrial function
Source: Theranostics. 2021 May 24;11(15):7294–307. doi: 10.7150/thno.56070 (PMC8210609; doi:10.7150/thno.56070)
Supplement: Supplementary file 1 — Supplementary figures. [file thnov11p7294s1.pdf]

Supplementary Figures

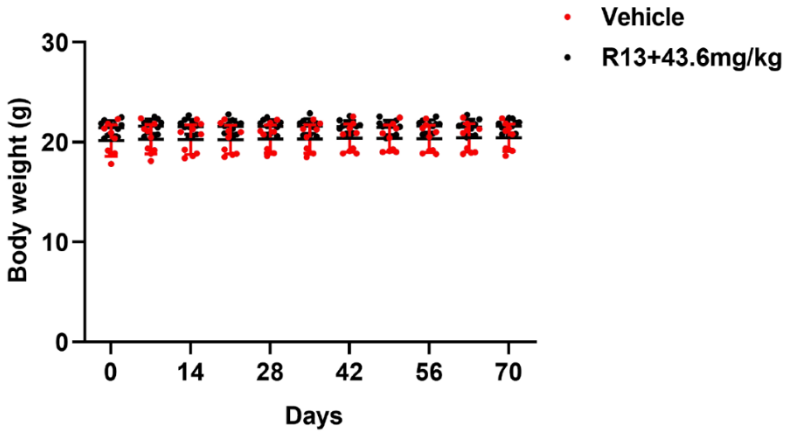

Figure S1. The effect of R13 treatment on the body weight of SOD1^{G93A} mice. The body weight of SOD1^{G93A} mice treated with 43.6 mg/kg R13 or wild type mice was detected.

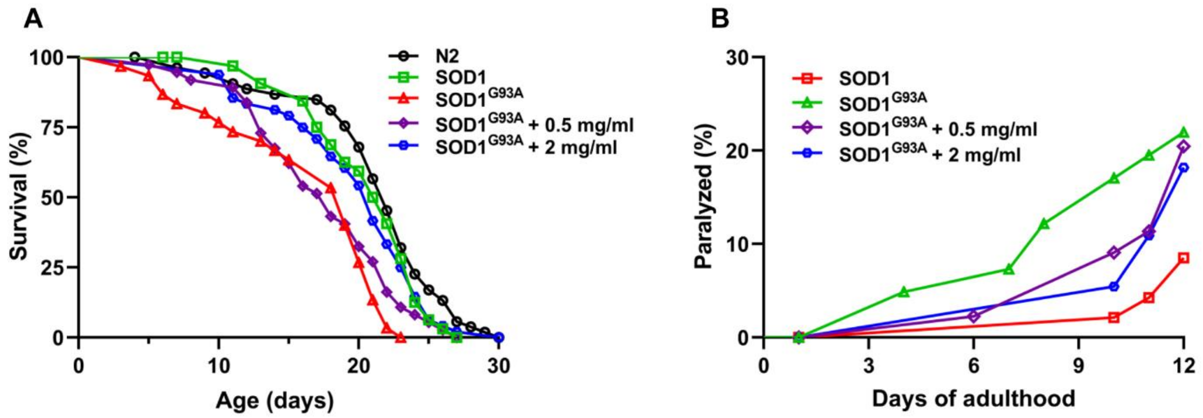

Figure S2. The effect of 7,8-DHF (active ingredient of R13) treatment on the survival rate and paralysis rate of worms stably expressing G93A SOD1. (A) 7,8-DHF significantly prolonged the survival rate of worms stably expressing G93A. **(B)** 7,8-DHF have no effect on the paralysis rate of worms stably expressing G93A SOD1.

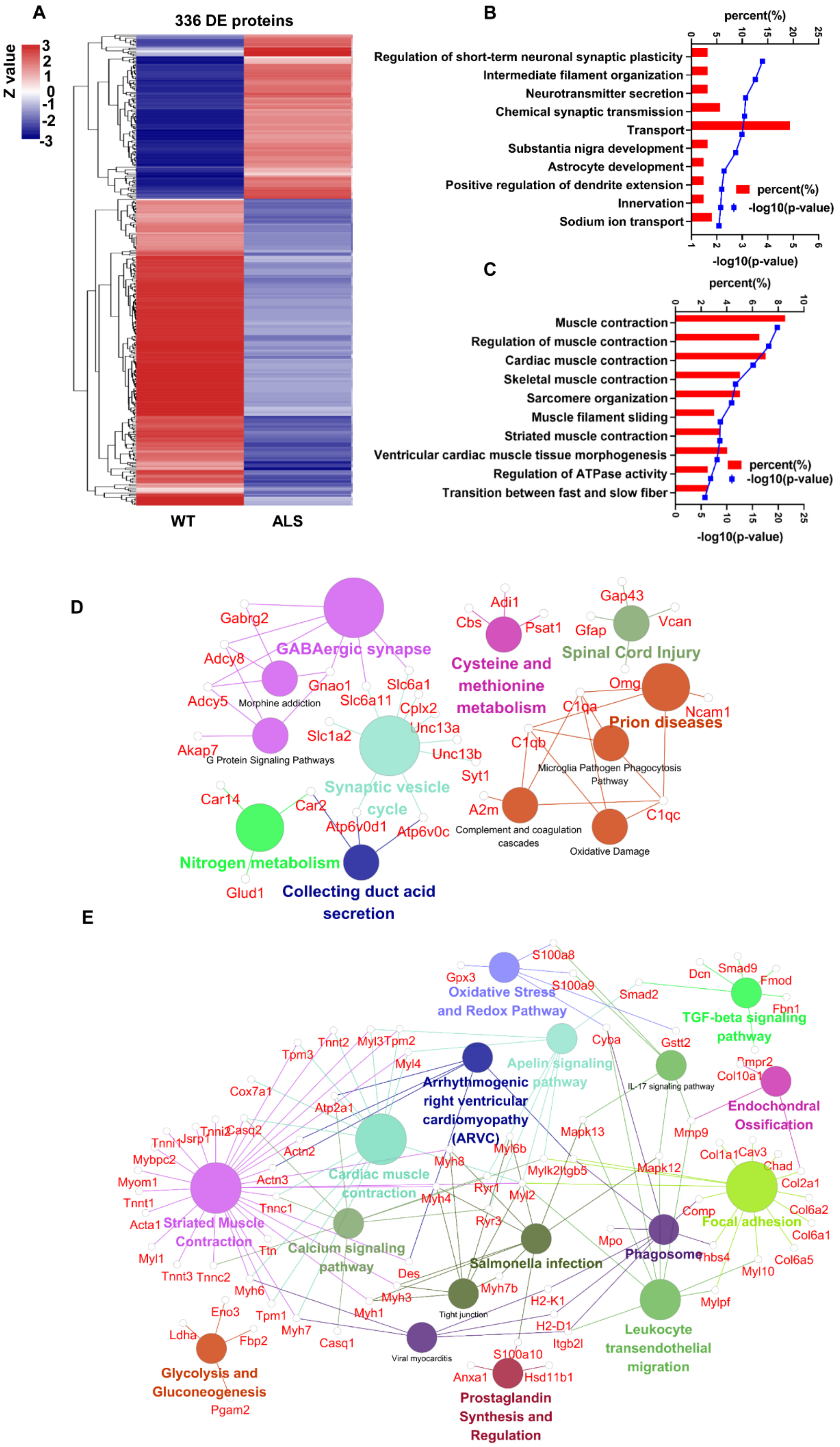
A
336 DE proteins
Z value
3
2
1
0
-1
-2
-3
WT
ALS
B
percent(%)
0
5
10
15
20
25
Regulation of short-term neuronal synaptic plasticity
Intermediate filament organization
Neurotransmitter secretion
Chemical synaptic transmission
Transport
Substantia nigra development
Astrocyte development
Positive regulation of dendrite extension
Innervation
Sodium ion transport
percent(%)
-log10(p-value)
1
2
3
4
5
6
-log10(p-value)
C
percent(%)
0
2
4
6
8
10
Muscle contraction
Regulation of muscle contraction
Cardiac muscle contraction
Skeletal muscle contraction
Sarcomere organization
Muscle filament sliding
Striated muscle contraction
Ventricular cardiac muscle tissue morphogenesis
Regulation of ATPase activity
Transition between fast and slow fiber
percent(%)
-log10(p-value)
0
5
10
15
20
25
-log10(p-value)
D
Gabrg2
GABAergic synapse
Adcy8
Adcy5
Morphine addiction
Gnao1
Akap7
G Protein Signaling Pathways
Slc6a1
Slc6a11
Cplx2
Unc13a
Slc1a2
Unc13b
Syt1
Synaptic vesicle cycle
Car14
Car2
Atp6v0d1
Atp6v0c
Nitrogen metabolism
Glud1
Collecting duct acid secretion
Adi1
Cbs
Psat1
Cysteine and methionine metabolism
Gap43
Gfap
Vcan
Spinal Cord Injury
Omg
C1qa
Ncam1
Prion diseases
C1qb
Microglia Pathogen Phagocytosis Pathway
A2m
C1qc
Complement and coagulation cascades
Oxidative Damage
E
S100a8
Gpx3
S100a9
Oxidative Stress and Redox Pathway
Smad2
Dcn
Smad9
Fmod
Fbn1
TGF-beta signaling pathway
Cyba
Gstt2
Apelin signaling pathway
IL-17 signaling pathway
Col10a1
Endochondral Ossification
Tnnt2
Myl3
Tpm2
Tpm3
Myl4
Arrhythmogenic right ventricular cardiomyopathy (ARVC)
Cox7a1
Atp2a1
Tnni1
Jsrp1
Tnni2
Casq2
Mybpc2
Myom1
Tnnt1
Acta1
Myl1
Tnnt3
Tnnc2
Striated Muscle Contraction
Actn2
Actn3
Tnnc1
Cardiac muscle contraction
Myl6b
Myh8
Myh4
Ryr1
Myl2
Mylk2
Itgb5
Mapk13
Mmp9
Mapk12
Col1a1
Cav3
Chad
Col2a1
Col6a2
Col6a1
Col6a5
Focal adhesion
Comp
Thbs4
Myl10
Mylpf
Ryr3
Calcium signaling pathway
Ttn
Mpo
Phagosome
Salmonella infection
Des
Myh7b
Myh3
Tight junction
Myh6
Eno3
Tpm1
Myh1
H2-K1
H2-D1
Itgb2l
Leukocyte transendothelial migration
Ldha
Fbp2
Myh7
Casq1
Viral myocarditis
S100a10
Anxa1
Hsd11b1
Prostaglandin Synthesis and Regulation
Glycolysis and Gluconeogenesis
Pgam2

Figure S3. Proteomics analysis of differential proteins in spinal cord of ALS mice compared with WT mice. **(A)** The heatmap expression profile of differential proteins in spinal cord. **(B)** Biological processes involved in up-regulated proteins. **(C)** Biological processes involved in down-regulated proteins. ClueGo analysis for up-regulated proteins **(D)** and down-regulated proteins **(E)**.

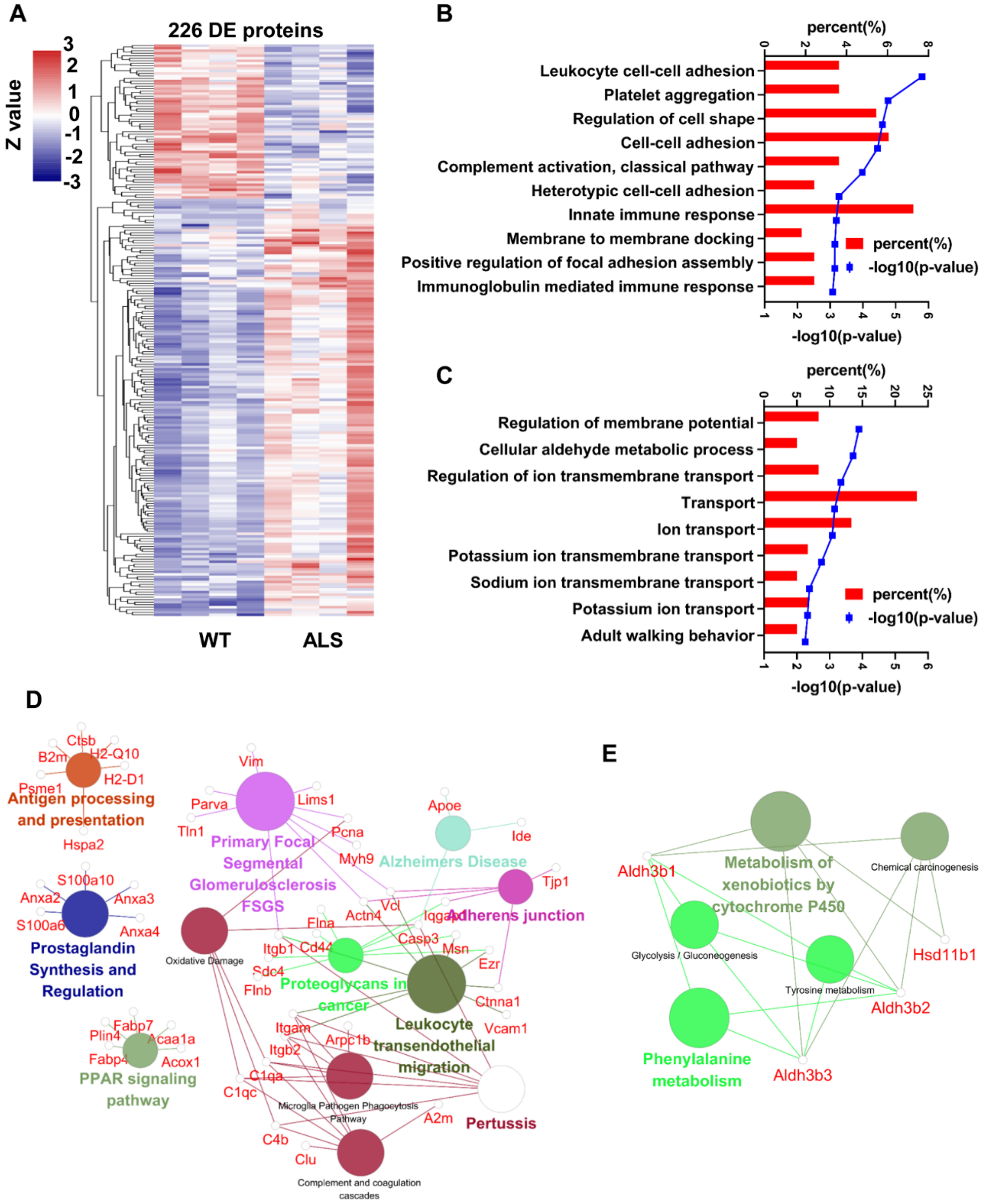

Figure S4. Proteomics analysis of differential proteins in medulla oblongata of ALS mice compared with WT mice. **(A)** The heatmap expression profile of differential proteins in medulla oblongata. **(B)** Biological processes involved in up-regulated proteins. **(C)** Biological processes involved in down-regulated proteins. ClueGo analysis for up-regulated proteins **(D)** and down-regulated proteins **(E)**.

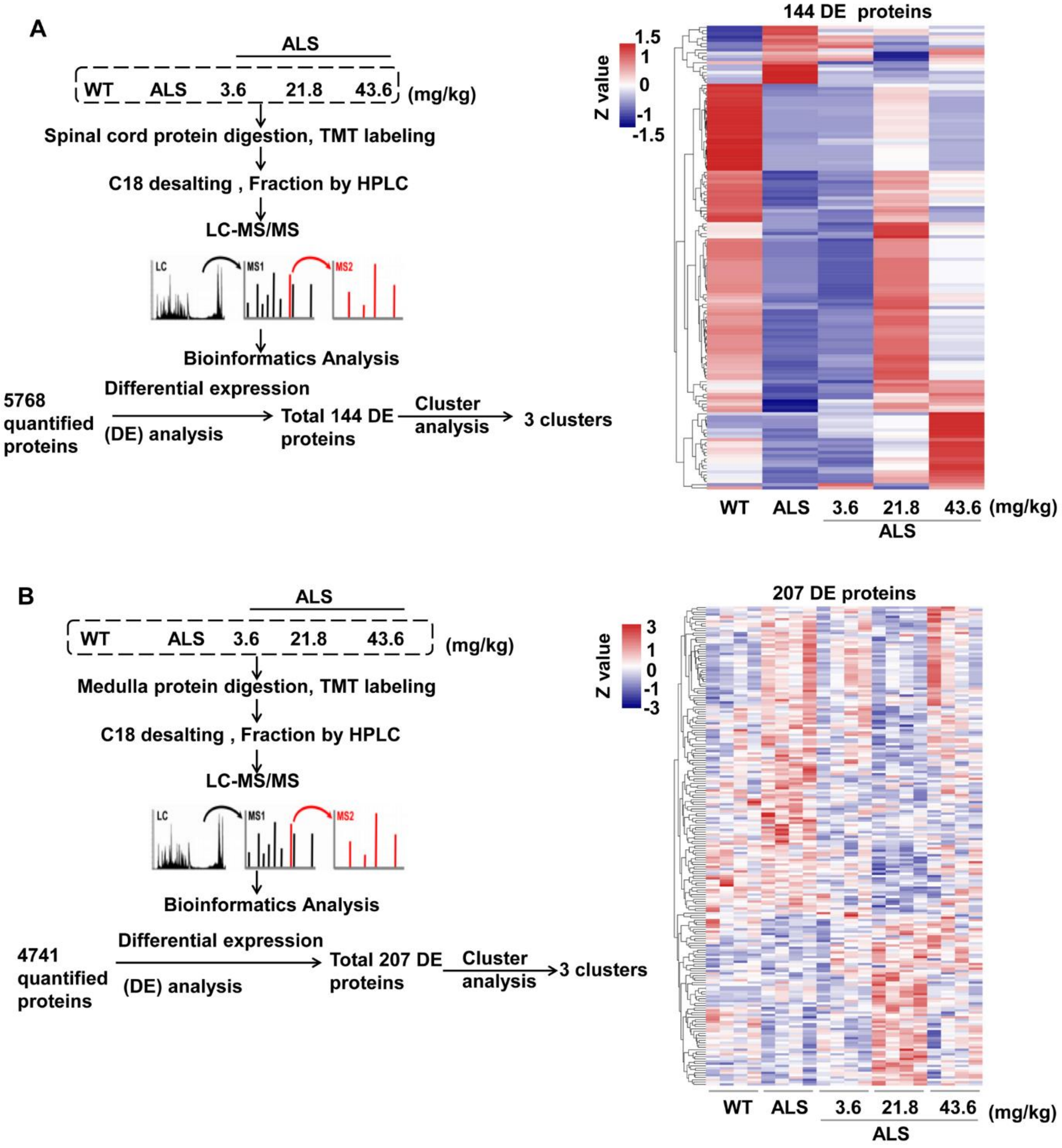

Figure S5. Short flow chart of proteomics, and heatmap analysis for differentially expressed proteins. (A) Spinal cord proteomics process and heatmap expression profile of differential proteins. **(B)** The proteomics process of the medulla oblongata and the heatmap expression profile of differential proteins.

A Spinal cord

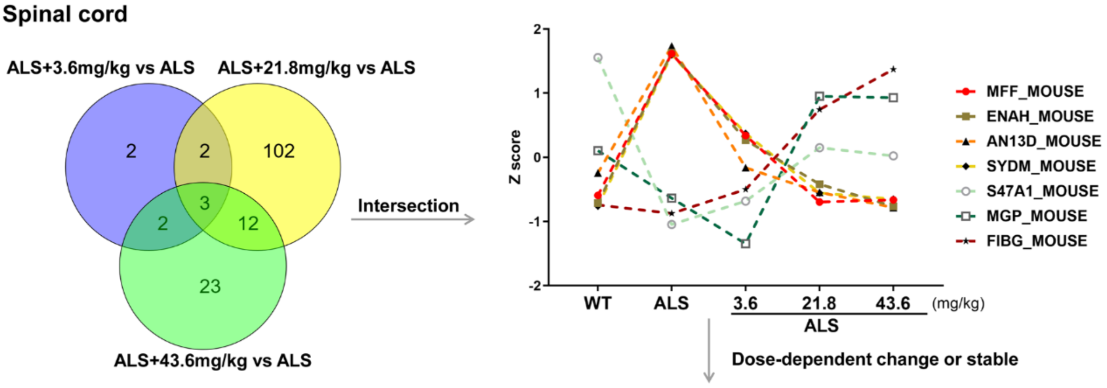

Uniport	Accession	Name	Gene	ALS vs WT	ALS+3.6mg/kg vs ALS	ALS+21.8mg/kg vs ALS	ALS+43.6mg/kg vs ALS
Q6PCP5	MFF_MOUSE	Mitochondrial fission factor	Mff	1.54	0.64	0.60	0.65
Q03173	ENAH_MOUSE	Protein enabled homolog	Enah	1.68	0.61	0.62	0.60
Q6PD24	AN13D_MOUSE	Ankyrin repeat domain-containing protein 13D	Ankrd13d	2.17	0.35	0.32	0.33
Q8BIP0	SYDM_MOUSE	Aspartate--tRNA ligase, mitochondrial	Dars2	0.47	1.35	3.32	1.98
Q8K0H1	S47A1_MOUSE	Multidrug and toxin extrusion protein 1	Slc47a1	0.43	1.26	2.77	1.54
P19788	MGP_MOUSE	Matrix Gla protein	Mgp	0.40	1.22	2.12	1.56
Q8VCM7	FIBG_MOUSE	Fibrinogen gamma chain	Fgg	0.80	1.03	1.50	1.62

B Medulla oblongata

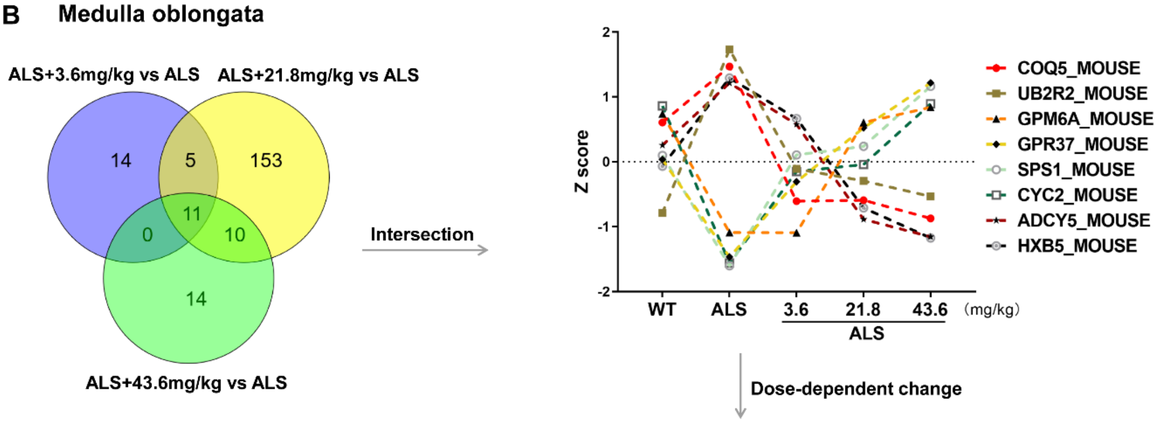

Uniport	Accession	Name	Gene	ALS vs WT	ALS+3.6mg/kg vs ALS	ALS+21.8mg/kg vs ALS	ALS+43.6mg/kg vs ALS
Q9CXI0	COQ5_MOUSE	2-methoxy-6-polyprenyl-1,4-benzoquinol methylase, mitochondrial	Coq5	1.18	0.78	0.76	0.77
Q6ZWZ2	UB2R2_MOUSE	Ubiquitin-conjugating enzyme E2 R2	Ube2r2	1.33	0.77	0.78	0.76
P35802	GPM6A_MOUSE	Neuronal membrane glycoprotein M6-a	Gpm6a	0.81	0.97	1.28	1.23
Q9QY42	GPR37_MOUSE	Prosaposin receptor GPR37	Gpr37	0.92	1.04	1.17	1.18
Q8BH69	SPS1_MOUSE	Selenide, water dikinase 1	Sephs1	0.94	1.03	1.11	1.11
P00015	CYC2_MOUSE	Cytochrome c, testis-specific	Cyct	0.86	1.05	1.10	1.15
P84309	ADCY5_MOUSE	Adenylate cyclase type 5	Adcy5	1.16	0.85	0.80	0.81
P09079	HXB5_MOUSE	Homeobox protein Hox-B5	Hoxb5	1.27	0.81	0.73	0.72

Figure S6. Dose-dependent analysis of differentially expressed proteins. (A) Venny analysis and dose-dependent analysis of differentially expressed proteins in spinal cord proteomic, and **(B)** The same analysis in medulla oblongata. The red box showed the mitochondrial protein.

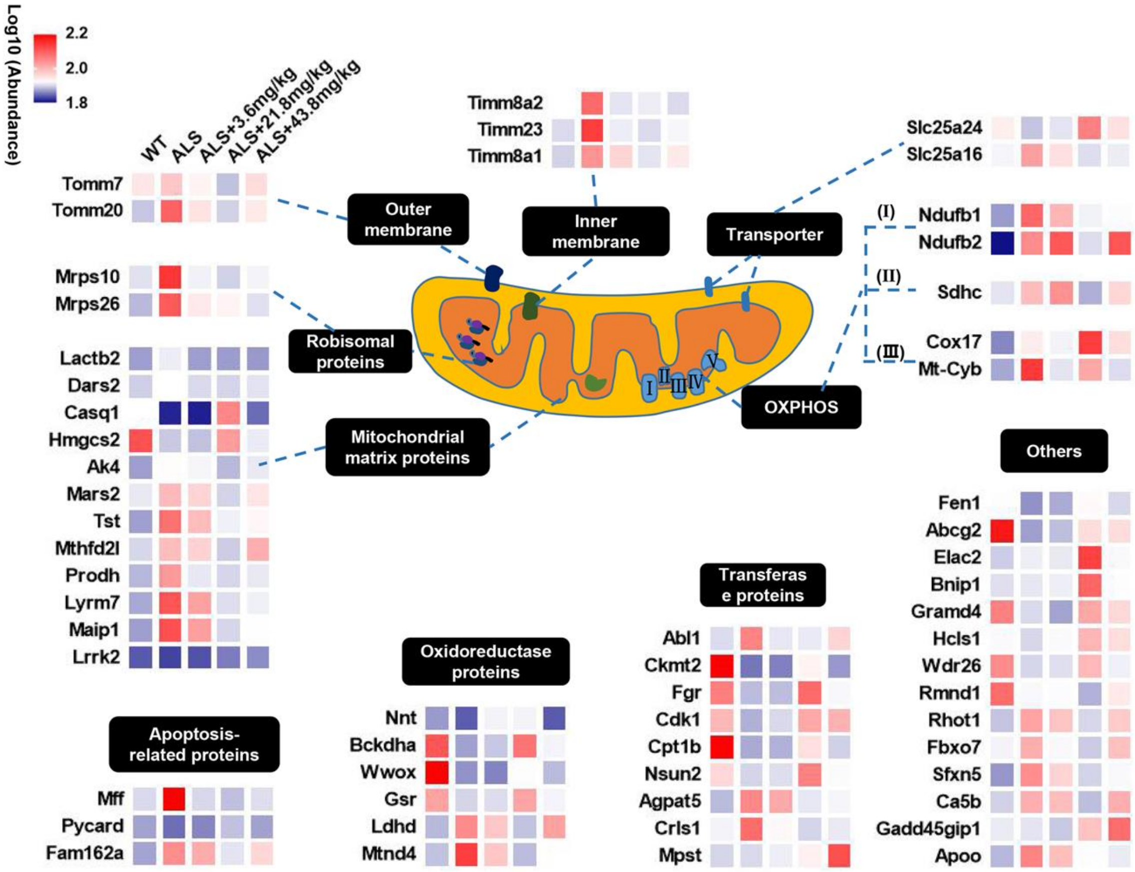

Figure S7. Differential expression profile of mitochondria in spinal cord. According to uniprot database and biological functions of GO annotations, differentially expressed proteins localized to mitochondria in spinal cord were mapped to apoptosis-related proteins, outer membrane, ribosomal proteins, mitochondrial matrix proteins, mitochondrial matrix proteins, oxidoreductase proteins, inner membrane, transporter, OXPHOS, transferase proteins, and others. The expression difference was set as ratio ≥ 1.2 or ≤ 0.83. The heatmap was color coded according to the log_{10} abundance for proteins. The color of blue represented low abundance, red represented high abundance.

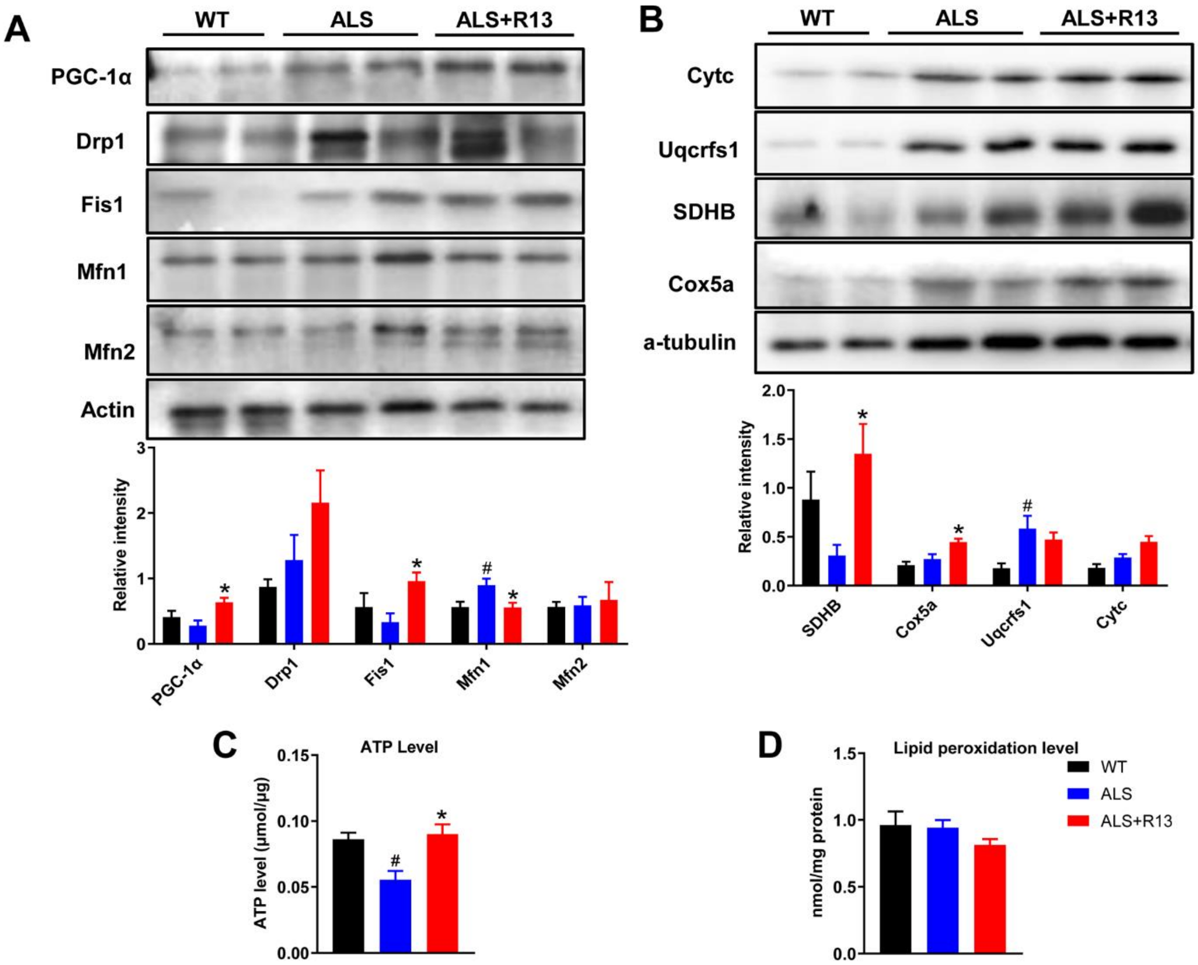

Figure S8. R13 treatment ameliorated mitochondrial function in spinal cord. (A) The expression of PGC-1α, Drp1, Fis1, Mfn1 and Mfn2, and **(B)** the electron transport chain proteins of Uqcrfs1, SDHB, Cox5a and Cytc were detected by Western blotting and quantitative analysis. **(C-D)** The levels of ATP and lipid peroxidation were detected. Data was expressed as Mean ± SEM. *, $p < 0.05$ vs. ALS vehicle group. #, $p < 0.05$, ##, $p < 0.01$ vs. WT group. n = 4 for each group.

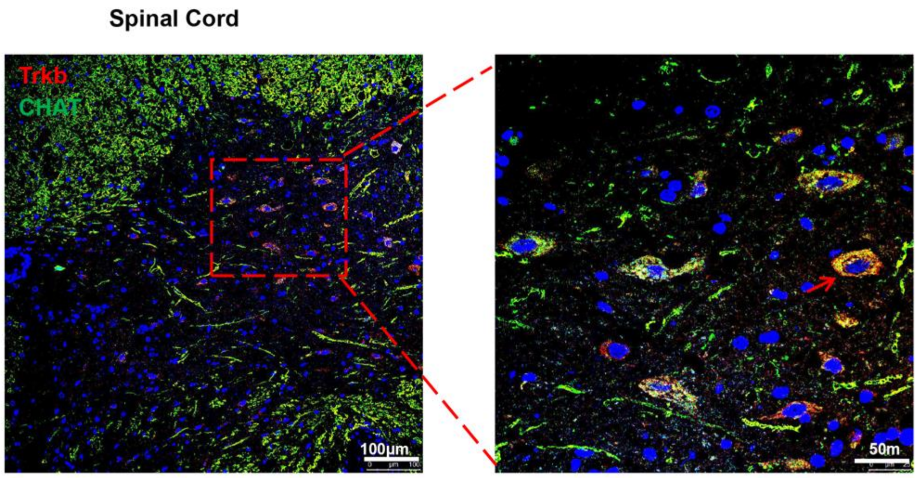

Figure S9. TrkB receptor localized in motor neurons of spinal cord. The representative image of co-localization of CHAT and TrkB receptors in motor neurons of spinal cord.

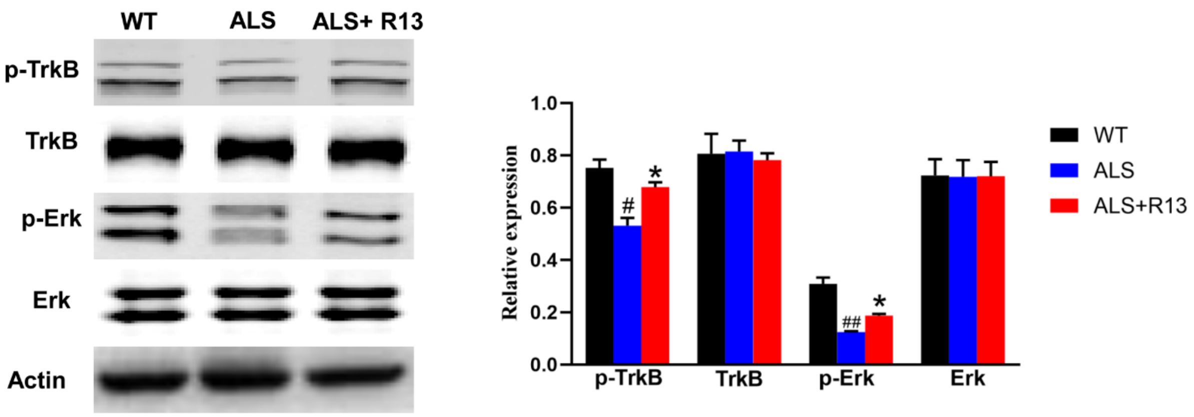

Figure S10. R13 activated the TrkB receptor signaling in motor cortex. The p-TrkB, TrkB, p-Erk and Erk were monitored by Western blotting and quantitatively analyzed. Data was expressed as Mean ± SEM. *, $p < 0.05$ vs. ALS vehicle group. #, $p < 0.05$, ##, $p < 0.01$ vs. WT group. n = 3 for each group.

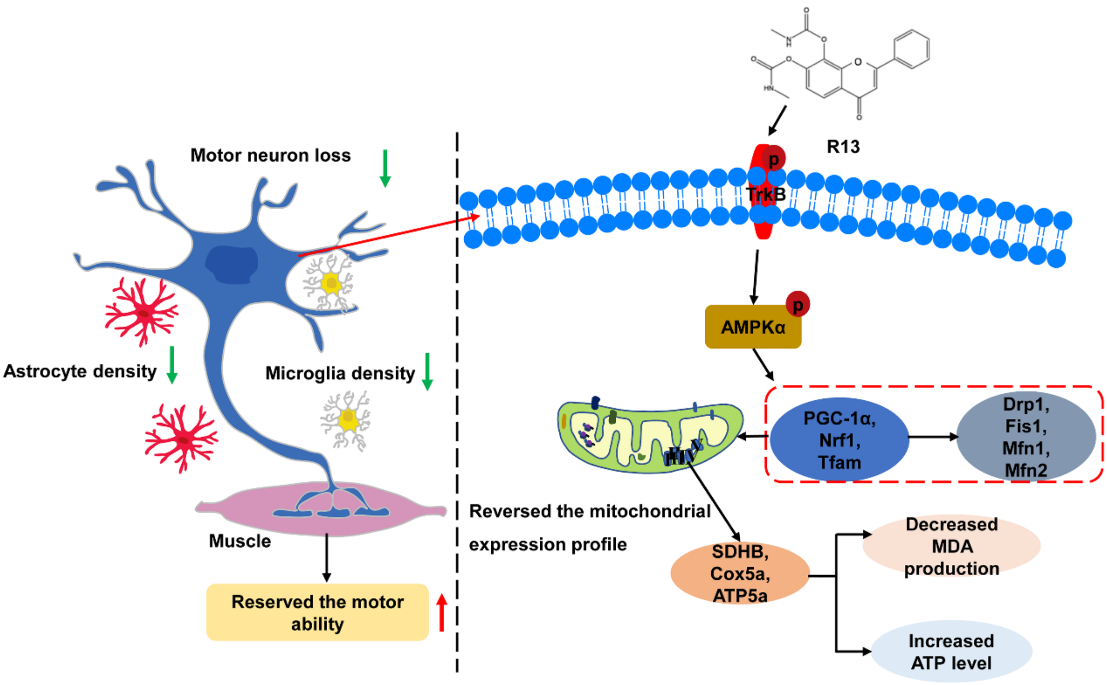

Figure S11. The possible mechanism of R13 in the treatment of SOD1^{G93A}. R13 activated AMPK to promote mitochondria biogenesis and fission via TrkB, and ameliorated mitochondrial proteins expression with increased ATP level and decreased lipid peroxidation level (MDA). R13 also suppressed the proliferation of glial cells, attenuated motor neurons loss, and ameliorated gastrocnemius function by upregulation of the proteins involving in striated muscle contraction pathway. All of these contributed to improve the motor ability of SOD1^{G93A} mice.

Supplementary Table 1 The dilution ratios and product information of antibodies

Antibody	Specificity	Type	Dilution	Source	CAT NO.
p-TrkB	Anti-phospho-TrkB (Tyr816) Antibody	Rabbit	1/500	sigma	ABN1381
TrkB	TrkB Antibody	Rabbit	1/1000	Biovision	3593
p-Akt	Phospho-Akt (Ser473)	Rabbit	1/1000	Cell Signaling	4060
Akt	Akt	Rabbit	1/1000	Cell Signaling	4691
p-AMPKα	Phospho-AMPKα (Thr172)	Rabbit	1/1000	Cell Signaling	2535
AMPKα	AMPKα	Rabbit	1/1000	Cell Signaling	2532
α-tubulin	Anti-Tubulin Antibody	Mouse	1/1000	Merck	MAB1637
PGC1-α	Anti-PGC1 alpha	Rabbit	1/1000	Abcam	ab54481
Nrf1	Anti-Nrf1	Rabbit	1/1000	Abcam	ab175932
Tfam	Anti-mtTFA	Rabbit	1/1000	Abcam	ab131607
Drp1	Drp1	Mouse	1/1000	Santa Cruz	sc-271583
Fis1	Fis1	Rabbit	1/1000	proteintech	10956-1-AP
Mfn1	Mitofusin 1	Mouse	1/1000	Abcam	ab57602
Mfn2	Mitofusin 2	Rabbit	1/1000	proteintech	12186-1-AP
Uqcrfs1	Anti-UQCRFS1/RISP	Rabbit	1/1000	Abcam	ab131152
ATP5a	Anti-ATP5A	Rabbit	1/1000	Abcam	ab14748
cytc	Cytochrome c	Rabbit	1/1000	Cell Signaling	11940
SDHB	Anti-SDHB	Mouse	1/1000	Abcam	ab14714
Cox5a	Anti-COX5A	Mouse	1/1000	Abcam	ab110262
Actin	β-Actin	Mouse	1/1000	Santa Cruz	sc-47778
